# Supplementary material for: The genome sequence of the fungus gnat, Macrocera phalerata Wiedemann in Meigen, 1818 (Diptera: Keroplatidae)
Source: Wellcome Open Res. 2026 Jul 10;11:431. [Version 1] doi: 10.12688/wellcomeopenres.26958.1 (PMC13562954; doi:10.12688/wellcomeopenres.26958.1)
Supplement: Peer review history

# Reviewer report: Reviewer response for version 1

**DOI:** 10.21956/wellcomeopenres.29679.r164567

**Published:** 2026-09-08

**Authors:** Richard P Meisel

**Affiliations:** 1 University of Houston, Houston, Texas, USA

The manuscript reports a standard genome assembly and annotation. It is sufficient for the purposes of a genome report. The lack of sex chromosome identification is understandable given the sex of the sample and the uncertainty about sex chromosomes in Sciaroidea. However, Keroplatidae is sister to Sciaridae, where sex chromosomes have been extensively studied. It may be possible to identify the X chromosome based on homology with Sciaridae species where the sex chromosomes have been identified.

Are sufficient details of methods and materials provided to allow replication by others?

Yes

Is the rationale for creating the dataset(s) clearly described?

Yes

Are the datasets clearly presented in a useable and accessible format?

Yes

Are the protocols appropriate and is the work technically sound?

Yes

Reviewer Expertise:

Evolutionary genomics

I confirm that I have read this submission and believe that I have an appropriate level of expertise to confirm that it is of an acceptable scientific standard.

# Reviewer report: Reviewer response for version 1

**DOI:** 10.21956/wellcomeopenres.29679.r166758

**Published:** 2026-09-03

**Authors:** Laurence Despres

**Affiliations:** 1 Université Grenoble Alpes, Auvergne-Rhône-Alpes, Grenoble, France

The authors report on the chromosome-level genome assembly of a female specimen of the fungus gnat, *Macrocera phalerata* (Diptera: Keroplatidae). The genome sequence has a total length of 572.81 megabases, scaffolded into 5 chromosomes. Sex chromosome(s) could not be identified as sequence data from the heterogametic sex was not available and homology is unreliable for sex chromosome identification in Diptera due to frequent sex chromosome turnover. The mitochondrial genome has also been assembled, with a length of 20.98 kilobases.  

 The genome assembly is of high quality and was generated using robust and appropriate methodologies, combining PacBio HiFi long reads (25× coverage) with Hi-C data to validate and improve chromosome scaffolding. BUSCO v.6.0.0 analysis using the diptera_odb10 reference set ( *n* = 3 285) identified 95.1% of the expected gene set (single = 92.8%, duplicated = 2.3%). The assembly and presentation of the results follow the high standards established by the Darwin Tree of Life Project. The BlobToolKit snail plot provides a comprehensive overview of the main assembly metrics and BUSCO gene completeness.

Are sufficient details of methods and materials provided to allow replication by others?

Yes

Is the rationale for creating the dataset(s) clearly described?

Yes

Are the datasets clearly presented in a useable and accessible format?

Yes

Are the protocols appropriate and is the work technically sound?

Yes

Reviewer Expertise:

evolutionary biology, population genomics

I confirm that I have read this submission and believe that I have an appropriate level of expertise to confirm that it is of an acceptable scientific standard.
